# Simultaneous Strength and Elongation Enhancement of Al-5Si Alloy and Welding Performance via Trace Cu/La Addition

**DOI:** 10.3390/ma19040730

**Published:** 2026-02-13

**Authors:** Wenwen Wu, Xianqi Meng, Sanxuan Han, Jingbo Liu, Xiaowei Lei, Nan Wang

**Affiliations:** 1 School of Physical Science and Technology, Northwestern Polytechnical University, Xi’an 710022, China; 2 Ningbo Institute of Northwestern Polytechnical University, Ningbo 315103, China; 3 Ningbo Boway Alloy High-tech Wires Co., Ltd., Ningbo 315135, China; 4 Western Superconducting Technologies Co., Ltd., Xi’an 710018, China; 5 Guiyang AECC Power Investment Casting Co., Ltd., Guiyang 550014, China

**Keywords:** Al-5Si alloy, Cu-La modification, mechanical properties, fluidity, welding performance

## Abstract

The addition of Cu or La plays an important role in microstructure and property manipulation of 4xxx series Al-Si alloys. However, the effects of Cu-La hybrid modification on the microstructure and properties of Al-5Si alloys and welding performance remain unclear. In this paper, the influence of Cu-La addition on the strength and elongation of one commercial Al-5Si alloy and the welding joint characterization are investigated. The results show that the addition of Cu-La can refine α-(Al) and Fe-rich phase and improve the fluidity. Meanwhile, the elongation can be improved by Cu-La microalloying, which is beneficial for the manufacturing filler wire. The uniform distribution of Cu in the alloy but not segregation at grain boundaries due to La addition is the key factor to adjust the mechanical properties. Moreover, the filler materials were used to conduct metal inert gas welding on 6061 alloy. It reveals that, with Cu-La addition, the weld pool width increases and porosity defect decreases significantly. This is ascribed to Cu-La co-addition enhancing wettability and fluidity, which improves the welding performance. Our results offer an effective strategy for manufacturing and optimizing welding performance of welding wires.

## 1. Introduction

Hypoeutectic Al-5Si alloys are extensively utilized as filler materials in the welding of new energy vehicles, owing to their high purity and superior welding efficiency [1,2]. Nevertheless, in practical applications, Al-5Si welding wires frequently exhibit high porosity in welded joints and unstable mechanical properties—issues primarily attributed to material composition and manufacturing processes. To enhance the comprehensive performance of this alloy system, modification treatments are commonly employed to refine the coarse eutectic Si phases [3,4], thereby improving mechanical properties.

Modification strategies for Al-Si alloys generally fall into two categories: rapid cooling modification achieved by increasing the melt cooling rate and chemical modification [5] involving the addition of specific elements during melting to alter the morphology and size of the eutectic silicon. However, constrained by fabrication processes, materials produced via rapid cooling fail to meet current industrial dimensional requirements and often exhibit significant internal residual stresses; consequently, this method has limited industrial application. As a result, the chemical modification has emerged as the prevailing approach for improving Al-Si alloy performance.

Hu et al. [6] employed Sm for the refinement of Al-Si alloys, observing that an addition of 0.6 wt.% Sm yielded peak ultimate tensile strength (276 MPa) and elongation (3.76%). Similarly, Rejaeian et al. [7] investigated the effect of Be addition on the microstructure of A380 aluminum alloy, noting a distinct refinement of the eutectic Si phase. Furthermore, Wang et al. [8] systematically explored the impact of Sc content on the microstructure and tensile properties of Al-Si alloys. Their findings revealed that Sc not only forms primary Al_3_Sc phases that serve as heterogeneous nucleation sites for α-Al grain refinement but also transforms the eutectic silicon from coarse platelets into fine fibrous structures. This optimization of Si morphology, combined with significant grain refinement, resulted in a marked enhancement of mechanical properties.

Although single-element modification yields positive results, it is associated with certain limitations, such as an increased susceptibility to porosity defects. Consequently, to overcome these drawbacks while achieving optimal comprehensive properties, research focus has shifted toward composite modification strategies. Qiu et al. [9] investigated the synergistic modification effects of La and Sr on Al-Si alloys. They observed that the modified eutectic Si tended to bend and split, resulting in a refined morphology. Furthermore, La atoms distributed at the solidification front of the Si phase and on dendrite surfaces acted as barriers to solute diffusion and exchange. This mechanism retarded solute accumulation on the silicon surface, reduced constitutional undercooling, and suppressed the growth rate of eutectic Si, thereby achieving simultaneous refinement of α-Al and silicon phases.

However, the aforementioned studies primarily focused on the microstructure and properties of as-cast Al-Si alloys, neglecting the influence of subsequent manufacturing processes involved in producing filler wires. Notably, the fabrication of 1.2 mm diameter Al-5Si filler wires requires multiple processing steps, including casting, extrusion, annealing, and drawing. This suggests that the inferior properties of the welding wires may be inherited from the preceding processing stages [10]. Thus, the factors influencing these different processing states must be considered to optimize the properties of the final filler products. Throughout this sequence, properties such as wettability, melt fluidity, tensile strength, and elongation exert a critical influence on processability. Our previous investigation [11,12] showed that copper can enhance the tensile strength of Al-5Si alloys through precipitation hardening, though often at the expense of elongation. Meanwhile, rare earth lanthanum (La) has been revealed to refine alloy grains and modify the eutectic silicon phase [13,14], thereby facilitating elongation control and optimizing comprehensive mechanical properties [15,16,17]. However, the effects of Cu-La hybrid modification on the microstructure and properties of Al-5Si commercial alloys and welding performance remain unclear.

In light of this, the present study utilizes a commercial Al-5Si-0.03Sr-0.05Ti alloy to investigate the synergistic mechanisms of Cu-La composite addition. Different from our previous investigated Al-5Si alloy [11,12], this commercial alloy itself contains Sr and Ti, which has the effect of grain refinement effect. The synergistic addition of Cu and La will be expected to improve the properties further for industrial application. The microalloyed alloys were cast, extruded, annealed, and drawn sequentially to ϕ1.2 mm filler materials. Furthermore, the filler wires were used in welding to evaluate the performance. The microstructure and mechanical properties of the alloys and the shape and the porosity of the weld bead are characterized and the effects of Cu-La microalloying are determined. It shows that Cu-La co-addition can improve the elongation to facilitate the manufacturing of the welding wires and increase the fluidity to enhance the welding performance. Our results can provide a deep insight into the mechanism of Cu-La microalloying and is helpful to develop new filler materials.

## 2. Experiment

### 2.1. Materials and Preparation

In this study, a commercial Al-5Si-0.03Sr-0.05Ti (sourced from Rio Tinto, Montreal, QC, Canada) alloy was employed as the base material (designated as Alloy 1). Alloy 2 was prepared by adding 0.05 wt.% Cu to the base alloy, while Alloy 3 was formulated by the further addition of 0.03 wt.% La to Alloy 2. The chemical compositions of the three alloys are listed in Table 1. All alloys were processed in a 3000 kg capacity non-vacuum natural gas-fired furnace (sourced from Wuxi Huguang Industrial Furnace Co., Ltd., Wuxi, Jiangsu, China).

The specific melting and fabrication procedures were as follows: Initially, 50% of the total pure aluminum charge was loaded into the furnace for melting, while the remaining 50% was reserved for temperature regulation. Once the initial aluminum charge had fully melted, Al-20Si, Al-50Cu, and Al-10La master alloys (sourced from Rio Tinto, Canada) were introduced. Subsequently, the reserved aluminum ingots were added to the melt to reduce the temperature to approximately 1073 K. The melt was then refined using a powder injection method with a commercial refining flux (sourced from Rio Tinto, Canada). After a holding period of 10 min, Al-10Sr and Al-5Ti-1B master alloys (sourced from Rio Tinto, Canada) were added, followed by an additional holding time of 5 min.

The melt temperature was adjusted to a range of 973–1003 K prior to casting to obtain as-cast samples (C-state). The material subsequently underwent continuous casting and rolling to produce rods with a diameter of 6.5 mm (F-state). These rods were subjected to recrystallization annealing at 643 K for 12 h to obtain annealed samples (O-state). The annealed rods were then drawn to a diameter of 3.5 mm to produce semi-finished wires (W-state). Finally, a secondary drawing process was employed to manufacture industrial-grade welding wires with a diameter of 1.2 mm (P-state).

### 2.2. Welding Application

**Figure 1:**
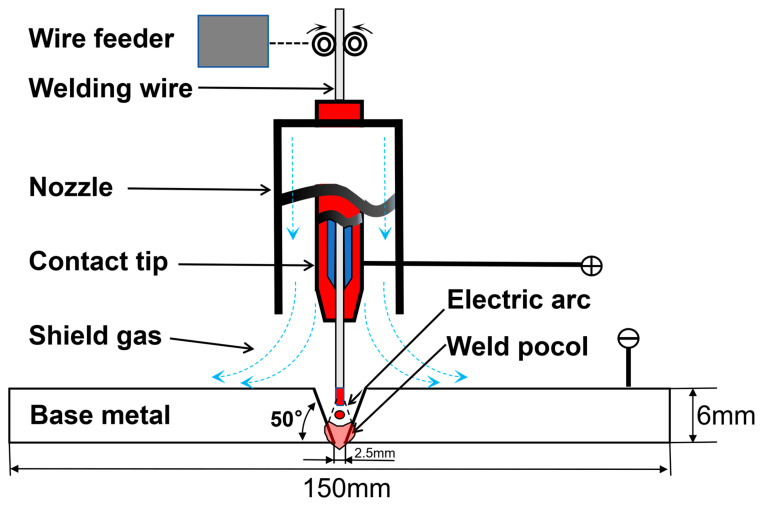
Schematic diagram of the MIG welding equipment and experimental setup.

To evaluate the influence of Cu-La composite micro-alloying on practical welding applications. Metal inert gas (MIG) welding experiments were conducted by the ϕ1.2 mm wires on base material 6061 alloy plates with dimensions of 330 mm × 150 mm × 6mm. The schematic diagram of the welding configuration is presented in Figure 1. A Fronius 600i welding power source was employed for the process. Based on standard industrial practices, the welding process parameters were selected as follows: welding current of 170 A, welding speed of 55 cm·min^−1^, torch angle of 70° to the horizontal, and 99.999% high-purity argon shielding gas at a flow rate of 20 L·min^−1^. To minimize experimental errors associated with high heat input and thermal instability, a two-pass welding strategy was adopted with strict temperature control. The substrate plates were first preheated to 373–413 K for 30 min prior to welding. Subsequently, the second pass was executed only after the interpass temperature had cooled to the range of 373–413 K. To ensure experimental reproducibility and eliminate contingency, three parallel experiments were performed for each condition using identical settings.

### 2.3. Microstructure Characterization

Following the welding experiments, specimens with dimensions ϕ15 mm × 5 mm for microstructural observation were prepared. The observation surfaces were mechanically polished and characterized in an unetched condition. Microstructural analysis and grain size distribution statistics were obtained using a Carl Zeiss upright optical microscope (OM; Zeiss, Oberkochen, Germany). A TESCAN S9000 GMH field-emission scanning electron microscope (SEM; TESCAN, Brno, Czech Republic) was utilized to analyze the weld morphology and elemental distribution. To characterize the morphology and elemental composition of precipitates, high-magnification imaging was performed using a Thermo Fisher Talos F200X transmission electron microscope (TEM; Thermo Fisher Scientific, Hillsboro, OR, USA) and a Themis Z double spherical aberration-corrected TEM (Themis Z TEM; Thermo Fisher Scientific, Hillsboro, OR, USA). Furthermore, to obtain accurate thermodynamic data for the optimization of processing parameters, differential scanning calorimetry (DSC) was conducted using a Discovery SDT simultaneous thermal analyzer (Discovery SDT; TA Instruments, New Castle, DE, USA). The distribution of porosity within the welded joints was examined using X-ray radiographic testing (RT-X; YXLON International, Hamburg, Germany). The inspection was conducted in accordance with the ISO 17636-1:2022 standard [18], utilizing an HS-XY-450/4549 X-ray inspection system.

### 2.4. Mechanical Properties Testing

Finally, to determine the key mechanical properties corresponding to different micro-alloying compositions, a series of wire and rod samples complying with dimensional specifications were prepared using a unified fabrication process. Tensile properties were evaluated using a computer-controlled electronic universal testing machine (UTM; Instron, Binghamton, NY, USA)at a crosshead speed of 5 mm·min^−1^. For the extruded, drawn, and annealed rod samples, the total length was 200 mm with a gauge length of 100 mm, and the testing diameter corresponded to the original diameter of the samples. For the as-cast samples, the gauge length was 100 mm, with a standard testing diameter of 10 mm.

## 3. Results

### 3.1. The Microstructure and the Mechanical Properties

**Figure 2:**
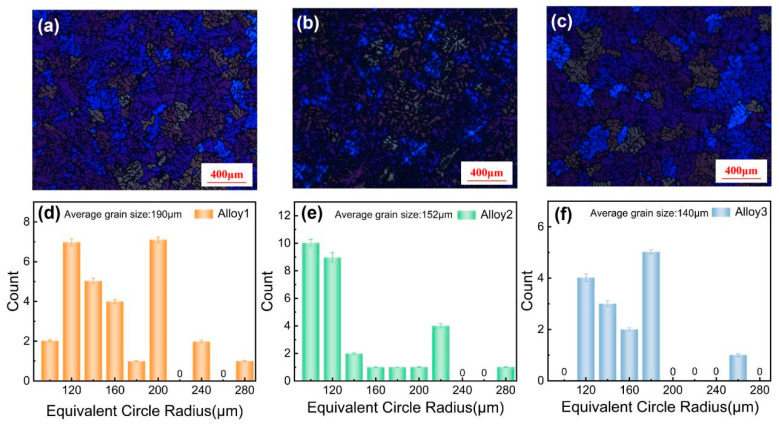
OM images and grain size distribution diagrams of the three alloys in the as-cast state. (**a**,**d**) Alloy 1 (**b**,**e**), Alloy 2, (**c**,**f**) Alloy 3.

Figure 2 presents the microstructural characterization of the three alloys in the as-cast (C) state. The low-magnification optical micrographs are displayed in Figure 2a–c, while the corresponding grain size distributions are shown in Figure 2d–f. As observed in Figure 2b, the addition of Cu resulted in a reduction in the grain size of Alloy 2 compared to Alloy 1. With the further addition of La, the grain size distribution of Alloy 3 became noticeably more concentrated, ranging primarily from 120 µm to 200 µm, with only 6.67% of grains exceeding 180 µm. In contrast, Alloy 2 exhibited a broader distribution range (100–220 µm), where the proportion of grains larger than 180 µm was 24.1%. These results indicate that Alloy 3 possesses a significantly more uniform grain structure than Alloy 2, alongside a further reduction in average grain size. Quantitative analysis performed using Image-Pro Plus software 6.0 determined the average equivalent circle diameters of Alloys 1, 2, and 3 to be 190 μm, 152 μm, and 140 μm, respectively (Figure 2d–f). This confirms the refinement efficacy of the Cu-La composite modification strategy.

**Figure 3:**
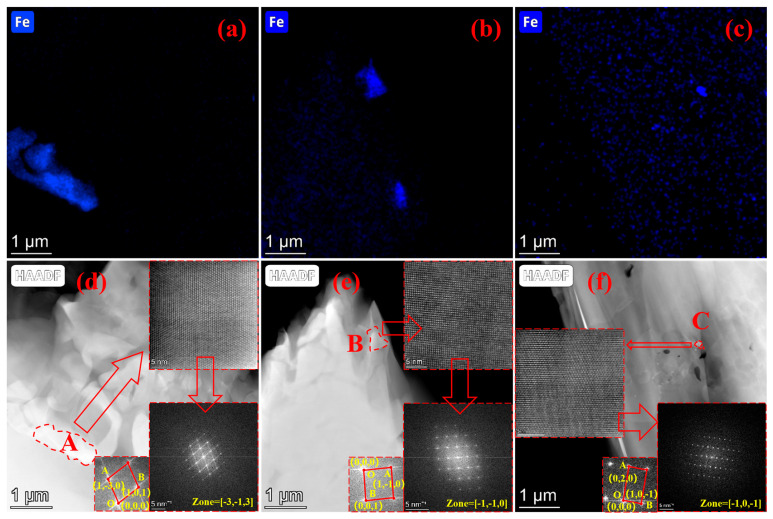
SEM/EDS characterization results and High-Angle Annular Dark-Field (HAADF) images and diffraction patterns of Fe-rich phase in as-cast state. (**a**,**d**) Alloy 1, (**b**,**e**) Alloy 2, (**c**,**f**) Alloy 3. A, B and C in the figure represent the Fe-rich phases of the three alloys, respectively.

In industrial Al-Si alloys, iron (Fe) is an unavoidable impurity that typically exerts detrimental effects on both processing and service performance. To investigate the mitigation of these effects, the morphology and composition of Fe-rich phases were characterized, as shown in Figure 3. High-Angle Annular Dark-Field (HAADF) images of the Fe-rich phases in the three alloys are presented in Figure 3a–c, with their corresponding Fe elemental distributions displayed in Figure 3d–f. In Alloy 1, the Fe-rich phase exhibits a coarse, blocky morphology. However, with the introduction of Cu and Cu-La, a distinct refinement in the size of these phases is observed. Fast Fourier Transform (FFT) analysis was conducted to identify the constituent phases. The results indicate that the Fe-rich phases in Alloys 1 and 2 correspond to the Al_5_SiFe phase, whereas, in Alloy 3, the phase transforms to Al_9_Si_3_Fe. This suggests that the addition of La effectively alters both the crystal structure and stoichiometric composition of the deleterious Fe-rich phases.

**Figure 4:**
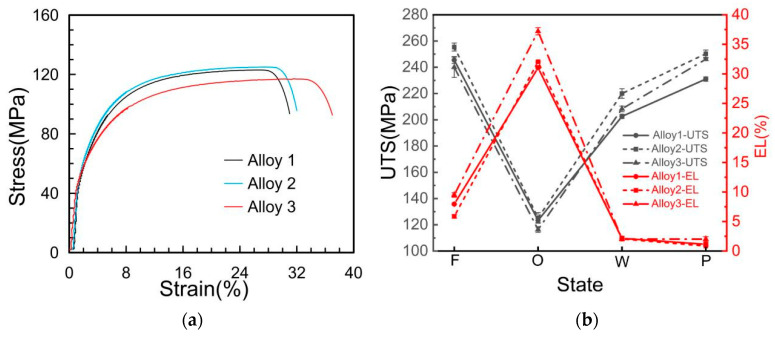
Mechanical properties of the three alloys. (**a**) Typical stress–strain curves of the three alloys in the annealed state; (**b**) determined ultimate tensile strength (UTS) and elongation (EL) of the three alloys in four states.

Figure 4 summarizes the mechanical properties of the three alloys across four processing states (F, O, W, and P), derived from tensile stress–strain curves. A representative stress–strain curve for the annealed (O) state is provided in Figure 4a, while the ultimate tensile strength (UTS) and elongation (EL) for all states are compared in Figure 4b. Similar trends in mechanical property evolution are observed across all three alloys; UTS initially decreases from the F-state to the O-state, then increases through the W- and P-states due to work hardening; conversely, elongation exhibits the opposite trend.

Crucially, even in the final work-hardened P-state, the elongation of the Cu-La-modified wire (Alloy 3) increased to 2%, compared to 1% for the Cu-only wire (Alloy 2). Simultaneously, the UTS increased from 231 MPa to 246 MPa. This simultaneous enhancement of strength and ductility in the final filler material confirms the benefits of Cu-La composite microalloying for practical welding applications.

### 3.2. The Characterization of Welding

**Figure 5:**
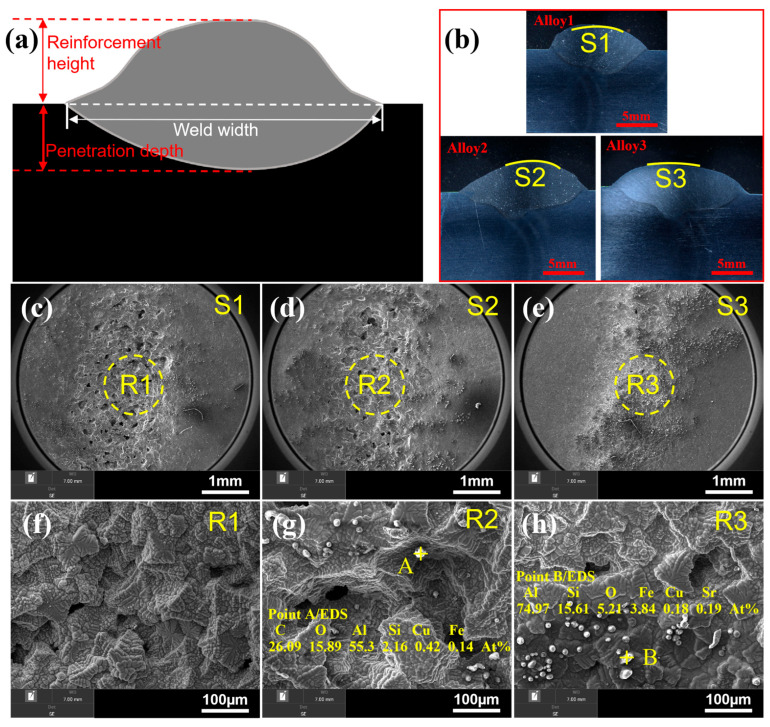
The shapes and microstructures of the weld bead by using the three alloys. (**a**) Schematic illustration of cross-sectional shape and parameters, (**b**) cross-sectional shapes of the three weld beads, (**c**–**e**) low magnification and (**f**–**h**) high magnification of surface morphologies of the three alloys.

To quantitatively evaluate the welding performance of the novel filler wires developed in this study, the geometric parameters of the weld beads—specifically weld width, reinforcement height, and penetration depth—were measured. A schematic illustration of these parameters is provided in Figure 5a, while the cross-sectional dimensions of the actual weld beads are presented in Figure 5b.

The results indicate that, following Cu microalloying, Alloy 2 exhibited increased weld width, reinforcement height, and penetration depth compared to Alloy 1. This enhancement could be partly due to the improved fluidity of the molten pool and partly due to the varied heat input resulting from the change in electrical conductivity by the Cu addition. With the subsequent addition of La, the weld width of Alloy 3 increased further, whereas the reinforcement height and penetration depth showed a slight reduction. The specific geometric measurements for all weld beads are summarized in Table 2.

Generally, a wider weld bead combined with reduced penetration depth contributes to a smoother joint profile and superior weld formation. Notably, after Cu-La composite modification, the weld width increased from 12.34 mm to 15.98 mm. This significant expansion demonstrates a marked improvement in the melt fluidity of the filler material, which is ascribed to the synergistic effects of Cu and La in reducing melt viscosity and modulating interfacial energy.

Subsequently, the surface morphologies of the weld beads produced by the three alloys were examined, as shown in Figure 5c–e. The weld surface of Alloy 1 appeared smooth and uniform. In contrast, the surface of Alloy 2 exhibited slight irregularities, while Alloy 3 displayed a further increase in surface roughness. High-magnification microstructural observations (Figure 5f–h) revealed the presence of grayish-white spherical particles on the bead surfaces of both Alloy 2 and Alloy 3, with a higher particle density observed in Alloy 3. Energy-Dispersive Spectroscopy (EDS) analysis conducted at point A in Figure 5g and point B in Figure 5h confirmed that these particles contain impurity elements, such as oxygen and iron.

**Figure 6:**
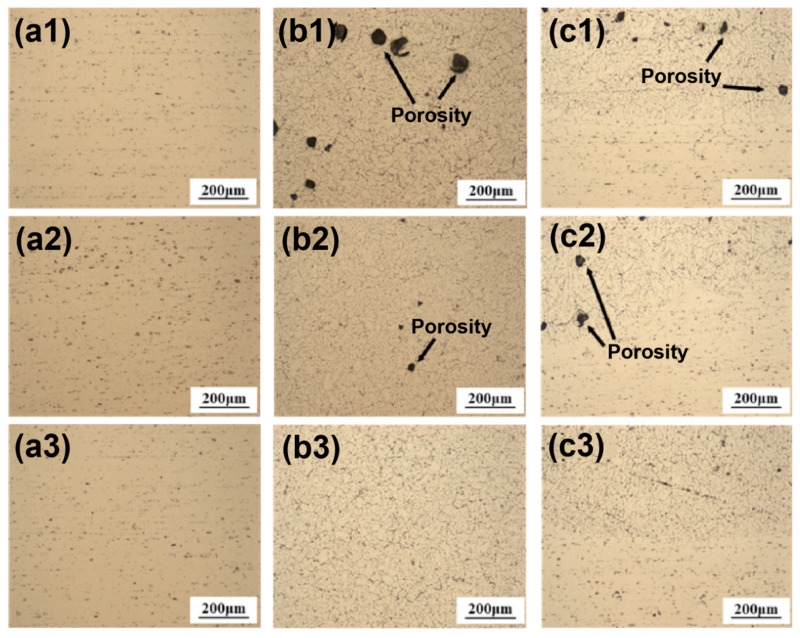
Optical micrographs (OM) of the welded joints produced using the three different filler wires: (**a1**–**a3**) base metal (BM); (**b1**–**b3**) fusion zone (FZ); (**c1**–**c3**) weld zone (WZ). Alloy 1: (**a1**–**c1**); Alloy 2: (**a2**–**c2**); Alloy 3: (**a3**–**c3**).

Finally, the microstructures of the welded joints produced by the three filler wires were characterized. Figure 6 illustrates the microstructures across the base metal, fusion zone, and weld zone for each joint. It is evident that the fusion zone of Alloy 1 (Figure 6(b1)) contains a substantial number of large-volume pores, whereas the fusion zone of Alloy 2 (Figure 6(b2)) exhibits only a few micropores. Furthermore, although the pore volumes in the weld zones of Alloy 1 (Figure 6(c1)) and Alloy 2 (Figure 6(c2)) are comparable, the pore population density in Alloy 1 is significantly higher than that in Alloy 2. In contrast, Alloy 3 displays no obvious porosity in either the fusion zone (Figure 6(b3)) or the weld zone (Figure 6(c3)).

**Figure 7:**
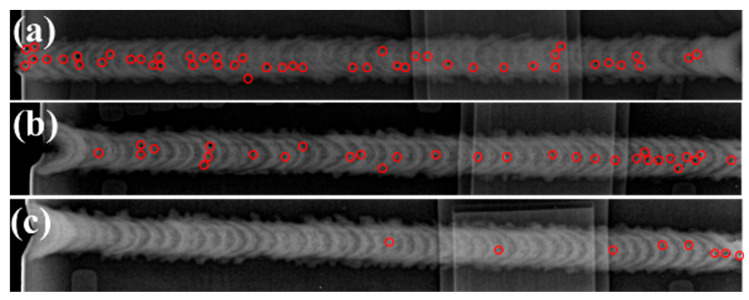
X-ray radiographic images showing porosity distribution in the welded joints produced by: (**a**) Wire 1 (Alloy 1); (**b**) Wire 2 (Alloy 2); (**c**) Wire 3 (Alloy 3).

To provide a macroscopic visualization of the porosity distribution and to elucidate the influence of the filler wires on defect formation, full-length X-ray radiographic inspection was performed on the weld seams. The results are presented in Figure 7, where the locations of detected pores are marked with red circles. Statistical analysis reveals that the Alloy 1 weld (Figure 7a) contained 48 pores, the Alloy 2 weld (Figure 7b) contained 31 pores, and the Alloy 3 weld (Figure 7c) contained only 8 pores. These findings demonstrate that the porosity in the weld produced by Wire 3 is significantly lower than that observed in welds produced by Alloys 1 and 2.

**Figure 8:**
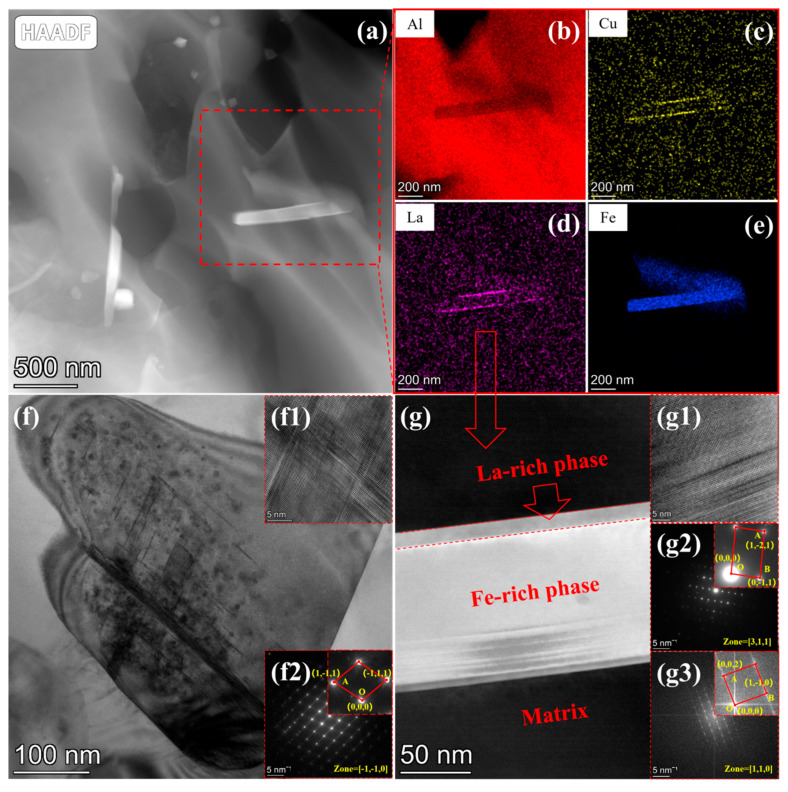
TEM images of weld bead by Alloy 3: (**a**) HAADF image and EDS mapping of (**b**) Al, (**c**) Cu, (**d**) La, and (**e**) Fe in the red dash square om (**a**), (**f**) The magnified HAADF image of the eutectic Si phase, (**f1**) is the HRTEM image of (**f**), (**f2**) is the SAED pattern of (**f**), and (**g**) The magnified HAADF image of the Fe-rich phase, (**g1**) is the HRTEM image of (**g**), (**g2**) is the SAED pattern of (**g**), (**g3**) is the FFT pattern of (**g**).

To further elucidate the influence of composite alloying elements on the welding process and joint properties, the internal microstructure of the Alloy 3 weld bead was analyzed, as shown in Figure 8. Figure 8a presents a high-angle annular dark-field scanning transmission electron microscopy (HAADF-STEM) image of the weld interior, where the dark contrast corresponds to the eutectic Si phase and the bright contrast identifies the Fe-rich intermetallic phases. STEM elemental mapping of the region marked by the red dashed square in Figure 8a reveals the distributions of Al, Cu, La, and Fe, which are displayed in Figure 8b–e, respectively. Notably, La exhibits a distinct sheath-like segregation around the Fe-rich phases. Figure 8f provides a magnified HAADF image of the eutectic Si phase shown in Figure 8a. A high-resolution TEM (HRTEM) image of this region (Figure 8(f1)) reveals the presence of abundant intersecting twins within the eutectic Si structure. Selected Area Electron Diffraction (SAED) analysis and indexing of this region confirm the phase as eutectic Si, as shown in Figure 8(f2).

Similarly, high-magnification HAADF imaging was performed on the Fe-rich phase (Figure 8g), revealing the presence of internal stacking faults. The corresponding HRTEM image of these stacking faults is presented in Figure 8(g1), while the SAED and Fast Fourier Transform (FFT) patterns of the Fe-rich phase are shown in Figure 8(g2) and 8(g3), respectively. The analysis identifies the Fe-rich phase in Region G as Al_9_Si_3_Fe. When combined with the phase identification results from Figure 3f, it is evident that the type of Fe-rich phase in the welding material remains unchanged after the surfacing process.

## 4. Discussions

### 4.1. The Effects on the Microstructures and Fe-Rich Phase in Cast State

As illustrated in Figure 2, the grain size significantly decreases with the addition of Cu and Cu-La. To elucidate the mechanism underlying this grain refinement, it is necessary to consider the variation in the nucleation energy barrier of the primary α-Al phase. It is established that the wetting angle (θ) is a critical parameter influencing the nucleation temperature and crystal growth kinetics, which, in turn, govern the nucleation energy barrier [19,20]. The critical free energy for heterogeneous nucleation (ΔG^*^_he_) is related to that of homogeneous nucleation (ΔG^*^_ho_) by the following relationship [19]:

(1) $$\Delta G_{he}^{*}=∆G_{ho}^{*}\;f(\theta )$$

and the wetting-angle factor *f* (*θ*) can be expressed as [21]:

(2) $$f(\theta )=\frac{27{(∆T)}^{2}\cdot T_{N}}{4T_{L}^{3}}=\frac{2-\cos\theta +{\cos\theta }^{3}}{4}$$

where *T_L_* and *T_N_* are liquidus and nucleation temperature and Δ*T* is undercooling.

**Figure 9:**
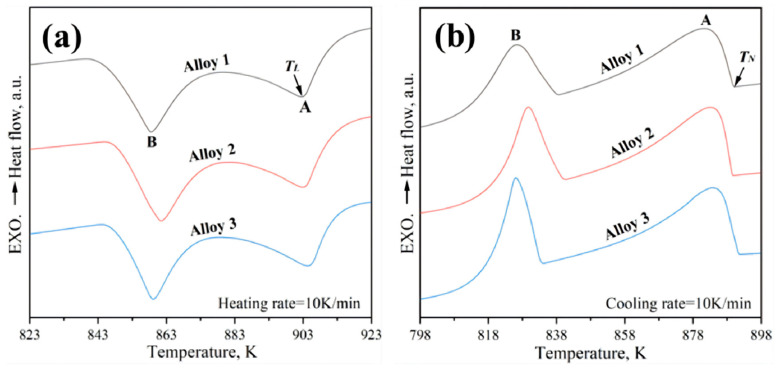
DSC curves of the three alloys in as-cast state. (**a**) Heating curves and (**b**) cooling curves.

To evaluate the effect of microalloying on the wetting angle and nucleation energy barrier, DSC analysis was performed on the three alloys; the results are presented in Figure 9. Based on the DSC heating and cooling curves, the liquidus temperature (*T_L_*), nucleation temperature (*T_N_*), and undercooling (Δ*T*) were determined and are summarized in Table 3. Using these experimental data, the wetting angle factor, *f* (*θ*), and the corresponding wetting angle (*θ*) were calculated via Equation (2) and are also listed in Table 3.

The calculated values of *f* (*θ*) for Alloys 1, 2, and 3 are 6.607 × 10^−4^, 5.724 × 10^−4^, and 5.113 × 10^−4^, respectively. The corresponding wetting angles are 14.1°, 13.5°, and 13.1°. These results indicate that the critical nucleation energy barrier for the heterogeneous nucleation of α-Al in Alloy 3 is reduced by approximately 29.22% compared to Alloy 1. Additionally, Alloy 3 exhibits the smallest undercooling. A reduced undercooling implies that solidification initiates at a higher temperature; consequently, the melt maintains a relatively higher temperature during the initial stages of solidification. This thermal condition helps sustain lower viscosity for a longer duration, thereby improving fluidity, which is highly beneficial for the welding process.

Regarding the impurity phases, Figure 3 demonstrates that the size of Fe-rich phases decreases significantly with Cu and Cu-La microalloying. Fe-rich phases typically precipitate at grain boundaries. As reported in previous studies [22,23], microalloying accelerates the nucleation and growth of these phases at the α-Al grain boundaries by inducing a wettability transition from complete to incomplete wetting. This mechanism effectively refines the Fe-rich phases. Furthermore, a reduction in the volume fraction and size of brittle Fe-rich phases significantly enhances the plasticity of the material, thereby facilitating the subsequent extrusion and drawing manufacturing processes.

### 4.2. The Effect on the Elongation and Ultimate Tensile Strength

The fabrication of commercial AlSi5 filler materials typically involves an initial extrusion of the cast ingot into a 6.5 mm rod (F-state), followed by a multi-stage drawing process to reduce the diameter first to 3.5 mm (W-state) and finally to 1.2 mm (P-state) for use as welding wire. The initial drawing stage from 6.5 mm to 3.5 mm imposes severe plastic deformation, requiring the material to possess not only adequate ultimate tensile strength (UTS) but also significant elongation to prevent fracture. Consequently, an intermediate annealing treatment is mandatory between the extrusion and drawing stages to restore ductility.

**Figure 10:**
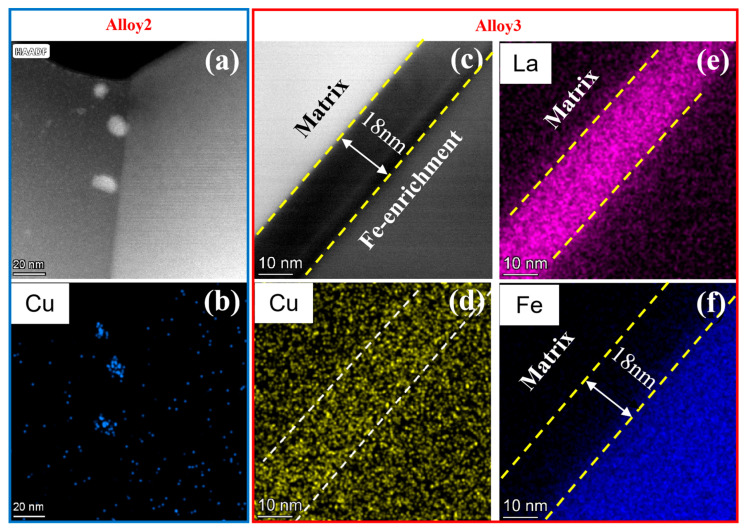
The STEM, HAADF, bright-field images at grain boundaries of Alloys 2 and 3. (**a**,**b**) Alloy 2, (**c**–**f**) Alloy 3 in the annealed state.

As illustrated in Figure 4, the annealing process (O-state) significantly increases the elongation of all three alloys, accompanied by a corresponding decrease in UTS. To elucidate the underlying mechanism governing these property changes, the elemental distribution of Cu and La at the grain boundaries of the microalloyed samples was investigated, with the results presented in Figure 10.

In the Cu-added Alloy 2, nano-scale Cu-rich phases are observed to precipitate primarily at the grain boundaries (Figure 10a,b). These intergranular precipitates effectively pin dislocations during deformation, thereby significantly enhancing strength but limiting ductility. In contrast, the grain boundaries in the Cu-La co-alloyed Alloy 3 are devoid of such coarse precipitates (Figure 10c). Instead, Cu is distributed more uniformly both within the grains and at the grain boundaries and is even detected within the Fe-rich phases. This distribution mitigates the strong dislocation pinning effect associated with grain boundary precipitates, resulting in improved elongation and a slight reduction in strength compared to Alloy 2.

Furthermore, La exhibits preferential segregation at the grain boundaries in Alloy 3, forming an approximately 18 nm thick La-rich shell at the interface between the α-Al matrix and the Fe-rich phase (Figure 10e,f). Previous studies suggest [17] that this segregation behavior is driven by the significant atomic size mismatch between Al and La, as well as the lower energy required for interfacial segregation compared to precipitation. This segregation behavior appears to suppress the precipitation of coarse Cu-rich phases at the grain boundaries while simultaneously promoting the nucleation of fine Cu-rich clusters within the matrix during solidification.

**Figure 11:**
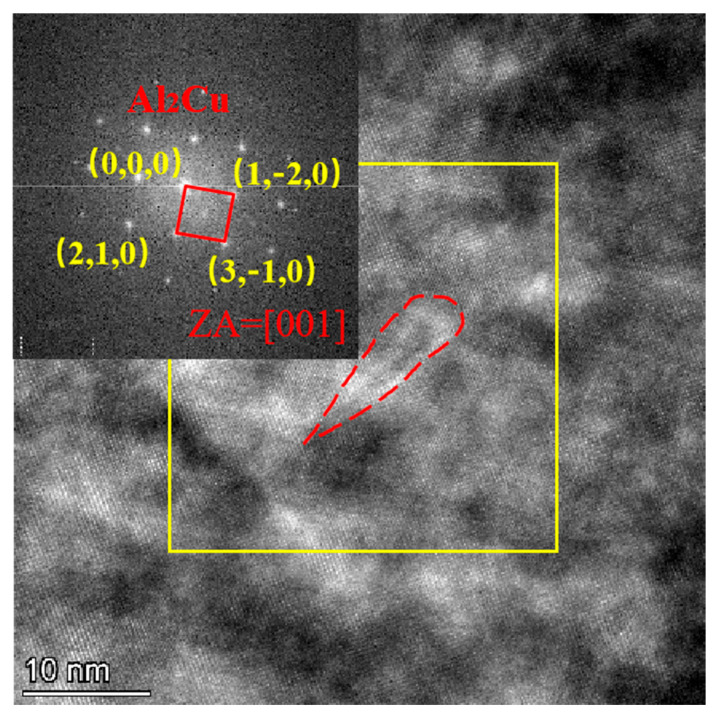
The HAADF image and selected area electron diffraction (SAED) pattern within grain of Alloy 3 in the annealed state.

The presence of these intragranular nano-scale Cu-rich phases (identified as Al2Cu) in Alloy 3 is confirmed in Figure 11. While these fine precipitates provide a considerable strengthening effect via precipitation hardening, they exert a lower dislocation pinning force compared to the coarser intergranular precipitates found in Alloy 2. Crucially, these fine intragranular phases are less detrimental to plasticity. As a result, Alloy 3 achieves a superior balance of mechanical properties, exhibiting higher elongation than Alloy 2 and maintaining higher strength than the base Alloy 1.

### 4.3. The Effects on the Welding

The results indicate that, following Cu-La composite addition, the weld width of Alloy 3 exceeds that of Alloys 1 and 2 (Figure 5b), signifying a significant enhancement in melt fluidity. Concurrently, impurity particles formed on the weld surface (Figure 5h) can be readily removed as slag during subsequent processing, which contributes to the long-term service performance and reliability of the welded joint. Furthermore, a statistical analysis of porosity within the weld seams reveals that Alloy 3 contains significantly less pores compared to Alloys 1 and 2. According to ISO 10042:2018 [24] radiographic standards, the excessive porosity in the Alloy 1 weld renders it non-compliant (unqualified). Alloy 2 meets Class C standards, making it suitable only for applications with lower quality requirements, whereas Alloy 3 demonstrates superior weld quality, suggesting a broader range of industrial applicability.

As illustrated in Figure 8, abundant intersecting twins are present within the eutectic Si phase of the Alloy 3 weld. Previous studies [25,26] indicate that such intersecting twins contribute to enhancing the mechanical properties of the material. Additionally, the stacking faults observed within the Al_9_Si_3_Fe phase are known to improve the material’s tear resistance under external loading. Consequently, these microstructural characteristics significantly enhance the comprehensive performance of the welded joint. Moreover, as this modification effect is intrinsic to the material’s metallurgy, it is likely applicable to other welding methods, indicating broad application prospects.

This study further elucidates the formation mechanism of the intersecting twins in eutectic Si and the stacking faults in the Fe-rich phase. Due to the significant atomic radius mismatch between La and Al, La preferentially segregates to the solidification front of the α-Al dendrites during the rapid cooling of the weld metal. Since Al-5Si is a hypoeutectic alloy, the nucleation temperatures of both the eutectic Si and AlSiFe phases are lower than the formation temperature of La-containing compounds. Furthermore, the thermal stress generated by the rapid cooling of the high-temperature molten pool, combined with the fast phase transformation within the weld, exerts compressive stress on the eutectic Si and Fe-rich phases. Under these stress conditions, interactional deformation occurs, ultimately leading to the formation of deformation twins and stacking faults.

## 5. Conclusions

In this study, a hypoeutectic Al-5Si welding filler material modified by Cu-La composite addition, featuring an exceptional balance of strength and ductility, was manufactured via continuous casting and rolling combined with precise alloying control. The results show that the addition of Cu-La can refine α-(Al) and Fe-rich phase, by which the elongation can be improved to facilitate the manufacturing of the filler wire. The La addition can lead to the uniform distribution of Cu in the alloy and plays a key role in adjusting the mechanical properties. The welding test reveals that, with Cu-La addition, the weld pool width increases and porosity defect decreases significantly. This is ascribed to Cu-La co-addition enhancing wettability and fluidity, which improves the welding performance. The alloy design strategy demonstrated in this study provides novel insights and technical support for the future development of high-performance hypoeutectic Al-Si welding materials.

## Figures and Tables

**Table 1:** Chemical composition list of the three alloys (wt.%).

Alloy	Si	Fe	Sr	Ti	Cu	La	Al
1	5.01	0.15	0.03	0.05	0	0	Rem
2	4.94	0.15	0.03	0.05	0.03	0	Rem
3	4.97	0.15	0.03	0.05	0.03	0.03	Rem

**Table 2:** Weld parameters of the three alloys.

Sample	Weld Width/mm	Penetration Depth/mm	Reinforcement Height/mm
Alloy 1	12.34	2.86	3.03
Alloy 2	14.50	3.19	3.25
Alloy 3	15.98	3.10	2.76

**Table 3:** Determined transition temperatures and the undercooling of α-(Al) phase.

Alloy No.	*T_L_* (K)	*T_N_* (K)	Δ*T* (K)	*f* (θ)	*θ* (°)
1	904.16	895.17	8.99	6.607 × 10^−4^	14.1
2	902.58	894.23	8.35	5.724 × 10^−4^	13.5
3	901.45	893.57	7.88	5.113 × 10^−4^	13.1

## Data Availability

The original contributions presented in this study are included in the article. Further inquiries can be directed to the corresponding author.
